# Quality of life in brain metastases radiation trials: a literature review

**DOI:** 10.3747/co.v15i5.290

**Published:** 2008-10

**Authors:** J. Wong, A. Hird, A. Kirou–Mauro, J. Napolskikh, E. Chow

**Affiliations:** * Rapid Response Radiotherapy Program, Department of Radiation Oncology, Odette Cancer Centre, Sunnybrook Health Sciences Centre, University of Toronto, Toronto, ON

**Keywords:** Brain metastasis, quality of life, whole-brain radiotherapy

## Abstract

**Background:**

An estimated 20%–40% of cancer patients will develop brain metastases. Whole-brain radiotherapy (wbrt) is the standard treatment for patients with brain metastases. Although wbrt can reduce neurologic symptoms, the median survival following wbrt is between 3 and 6 months. Given this limited survival, it is important to consider quality of life (qol) when treating patients with brain metastases. However, few studies have focused on qol and improvement in patient-rated symptoms as primary outcomes.

**Objective:**

For an accurate measurement of the extent to which previous trials have utilized qol tools to evaluate the efficacy of wbrt for treatment of brain metastases, we undertook a literature review to examine the common endpoints and qol instruments used.

**Methods:**

We conducted a systematic search using the medline (1950 to December 2007) and Cochrane Central Register of Controlled Trials (4th quarter 2007) databases. Eligible studies investigated wbrt in one of the study arms. The following outcomes were included: median survival, overall survival, neurologic function, 1-year local control, and overall response; use of qol instruments, performance status scales, and neurologic function assessments; and use of other assessment tools. Patient-rated qol instruments were defined as those that strove to assess all dimensions of qol; observer-rated performance instruments such as the Karnofsky performance status (kps) were deemed to be performance scales.

**Results:**

We identified sixty-one trials that included wbrt as a treatment for brain metastases. Of these sixty-one trials, nine evaluated the treatment of a single brain metastasis, and fifty-two evaluated the treatment of multiple brain metastases. Although fifty-five of the trials employed a qol instrument, few trials focused on qol as an outcome. We found 23 different instruments used to evaluate qol. The most commonly employed instrument was the kps (*n* = 33), followed by various neurologic function classification scales (*n* = 21). A preponderance of the studies used 1 (*n* = 26, 43%) or 2 (*n* = 21, 34%) qol instruments.

A total of fourteen published trials on brain metastases included an evaluation of the study population’s qol. Those trials included three that used the Functional Assessment of Cancer Therapy–General scale and Brain subscale instrument, three that used the European Organization for Research and Treatment of Cancer Quality of Life Questionnaire (C30) and the Brain Cancer Module 20 instrument, two that used study-designed qol instruments, one that used the Edmonton Symptom Assessment Scale, two that used the Spitzer Quality of Life index, and three that used the kps to evaluate qol. Some trials reported deterioration in qol after wbrt in patients with poorer prognosis; other trials detected an improvement in qol after wbrt in patients with better prognosis.

**Conclusions:**

To date, fourteen trials in brain metastases that have included an evaluation of the study population’s qol have been published. Although some studies showed that certain parameters of qol deteriorate after wbrt, other studies showed that qol in patients with better prognosis is improved after wbrt. Because a standard, validated qol instrument has not been used for this patient population, a comparison of findings concerning qol between the studies is difficult. The present review emphasizes the need to include qol measures in future wbrt clinical trials for brain metastases.

## 1. INTRODUCTION

Brain metastases are a cause of significant morbidity. An estimated 20%–40% of cancer patients will develop brain metastases during their illness 1. The most common primary cancers that metastasize to the brain are lung, breast, and gastrointestinal cancers 2,3. Depending on the location of the brain metastases, patients may suffer from neurologic symptoms that include headaches, focal weakness, mental disturbances, behavioural changes, seizures, speech difficulty, and ataxia 4. The prognosis for patients with brain metastases is generally poor; median survival is 1 month for patients not receiving treatment. Use of corticosteroids to reduce cerebral edema has been associated with symptom improvement 2.

Whole-brain radiotherapy (wbrt) is the standard treatment for brain metastases. About 30%–40% of affected patients present with a single brain metastasis, but most present with multiple lesions 5. The objective of wbrt is to provide symptomatic relief, to allow for tapering of the dose of corticosteroids, and to possibly improve survival. Although many trials have shown that wbrt can reduce neurologic symptoms, median survival following a diagnosis of brain metastases is generally only 3–6 months 6. Patients with a solitary brain metastasis, good performance status, and controlled extracranial disease may be considered for more aggressive treatment such as surgery with postoperative radiotherapy or stereotactic radiosurgery. Radiosensitizers, chemotherapy, and various radiotherapy dose fractionation schedules have also been explored to improve the outcome of brain metastases 7–11.

The World Health Organization (who) describes health as a “state of complete physical, mental, and social well-being and not merely the absence of disease or infirmity” 12. This subjective, multidimensional definition of health encourages health care professionals to focus not only on a patient’s length of life, but also his or her quality of life (qol). Quality of life can be seen as a balance between minimizing treatment risks and maximizing benefits, including physical and psychological effects 13. Because patients with brain metastases have limited survival, treatment options that are less morbid and that maximize qol are essential.

An Outcomes Working Group was formed by the Health Services Research Committee in the American Society of Clinical Oncology to define the outcomes of cancer treatment that should be considered for assessment and cancer treatment guidelines. Quality of life was rated as an endpoint secondary in importance only to survival. The group suggested that these two patient outcomes—survival and qol—should take precedence over cancer outcomes such as response rate^14^. The importance of including qol as a component of treatment assessment was also emphasized by Tannock, who wrote, “When cure is elusive, it is time to start treating the patient and not the tumor” 15.

Previous clinical trials have defined the efficacy of treatment using some of the following endpoints: survival, response, radiologic or imaging response, observer-rated neurologic symptoms, time to recurrence of intracranial disease, cause of death, and preservation of the ability to function independently 16–21. However, few studies have focused on qol and improvement in patient-rated symptoms as primary outcomes.

To accurately measure the extent to which previous trials utilized qol tools to evaluate the efficacy of radiotherapy for treatment of brain metastases, we undertook a literature review to examine the common endpoints and qol instruments used.

A preponderance of the published trials used a performance status scale such as the Karnofsky performance status (kps) to quantify the general well-being of patients with brain metastases 19,22–25. The purpose of a performance status assessment is to quantify a patient’s level of function, level of ambulation, and ability for self-care^26^ The kps is rated in increments of 10 on a scale of 0 to 100, with 100 meaning “normal, no complaints, no signs of disease” and 0 meaning “death”. A score is assigned based on assessment by an observer such as a physician, nurse, or researcher 27. Trials often use a performance score to describe their study population or as a component of the study’s exclusion criteria—for example, patients below a certain kps score are deemed ineligible 28,29.

Although performance status is one of the dimensions of qol, qol is subjective and should reflect how a patient feels 30. The kps was evaluated previously, and although it was found to be a reliable instrument, it did not capture the overall concept of qol 31–33. In the present study, only patient-rated instruments that strive to assess all dimensions of qol were deemed to be qol instruments; observer-rated performance instruments such as the kps were deemed to be performance scales.

## 2. METHODS

### 2.1 Search Strategy

We conducted a systematic search of the medline (1950 to December 2007) and Cochrane Central Register of Controlled Trials (4th quarter 2007) databases. The terms “brain neoplasms” and “brain metastas#s” were used. The subheading “secondary” was selected to narrow the search to metastases to the brain (so as to exclude primary brain cancer). That search was combined with the terms “radiotherapy” or “quality of life.” Relevant articles and abstracts were reviewed, and the reference lists from these sources were manually searched for additional relevant trials. The search was not limited by year of publication.

### 2.2 Inclusion Criteria

Articles were included in the literature review if they met these criteria:

- **Population** Studies of adult participants who had been diagnosed with one or more brain metastases by computed tomography or magnetic resonance imaging.
- **Intervention** wbrt in one study arm.
- **Type of study** Randomized or quasi-randomized trials and prospective or retrospective cohort studies.
- **Outcomes** Survival, qol, symptom control, neurologic function, toxicity, response of brain metastases to treatment, cause of death, duration of functional independence, and intracranial progression-free duration.

### 2.2 Exclusion Criteria

Articles were excluded from the literature review if they were

- individual case reports or review articles
- published in languages other than English, or
- phase i and ii trials for which phase iii trials were already available.

### 2.3 Data Extraction

The following information extracted from the studies:

- Number of patients accrued and evaluated in each study arm
- Patient inclusion and exclusion criteria for studies that included chemotherapy, surgery, or radiosurgery in one study arm
- Treatment details such as chemotherapy drugs or radiosensitizer
- Total dose and fractionation schedule for wbrt trials
- Outcomes such as median survival, overall survival, neurologic function, 1-year local control, and overall response
- Use of qol instruments, performance status scales, and neurologic function assessments
- Other assessment tools, if used

## 3. RESULTS

We identified sixty-one trials that treated patients using wbrt in at least one study arm.

### 3.1 Single Brain Metastasis

Nine of the sixty-one studies evaluated treatment of patients with a solitary brain metastasis. Two published trials and one abstract examined the role of surgery and wbrt as compared with wbrt alone 23,29,34. One trial assessed the effectiveness of surgery and wbrt as compared with surgery alone 19. Epstein *et al.*^35^ compared survival outcomes of various dose escalation schedules. One multi-institutional retrospective study investigated the use of radiosurgery and wbrt 36. A study by Jyothirmayi *et al.* 37 examined the efficacy of radiosurgery at diagnosis, radiosurgery and wbrt at diagnosis, and radiosurgery at recurrence. Another study compared the outcomes of radiosurgery alone, wbrt alone, and radiosurgery with wbrt 38 Roos *et al.* 39 investigated the results of randomizing patients to wbrt or observation after the patients had been treated with surgery or radiosurgery. Their study also examined the qol of the study population.

### 3.2 Multiple Brain Metastases

We identified fifty-two studies involving treatment of multiple brain metastases. One trial examined the use of corticosteroids and wbrt as compared with wbrt alone 40. In another trial, all patients received dexamethasone before wbrt, after which they were randomized to wbrt with a dose of dexamethasone or to wbrt alone 41. Two retrospective trials examined the outcomes of multiple treatments including wbrt, surgery, chemotherapy, or supportive care 42,43. Twelve studies examined the use of various wbrt dose fractionation schedules 6,10,22,28,44–51, and seven trials assessed the efficacy of radiosensitizers and wbrt as compared with wbrt alone 9,21,24,25,52–54. Chemotherapy and wbrt were compared in eight studies 7,8,55–60. Five trials examined the efficacy of whole-brain re-irradiation in patients with brain metastases 61–65. One study randomized patients with 1–3 brain metastases to wbrt or wbrt followed by stereotactic radiosurgery boost 18. One retrospective study examined the outcomes of wbrt or Gamma Knife radiosurgery 66. One randomized trial examined the combination of wbrt and radiosurgery as compared with wbrt alone for patients with 2–4 brain metastases 20. Three other studies looked at wbrt and radiosurgery as compared with radiosurgery alone for patients with 1–3 brain metastases 67, 1–4 brain metastases 68, and single or multiple brain metastases 69. One study focused on qol and the patients’ perspectives regarding management-related complications in addition to the radiosurgery 68. Another study investigated the survival and qol of patients who were randomized to wbrt with efaproxiral or to wbrt alone^70^. Six other studies examined the role of qol or patient-rated symptoms when assessing the effectiveness of wbrt 17,71–75. Two studies assessed the neurocognitive function (ncf) of patients who had been treated with wbrt and a radiosensitizer 76 or with wbrt alone 27. Lastly, one study investigated both ncf and qol of patients treated with wbrt 77.

### 3.3 Study Outcomes

Tables I–IX present the outcomes of the trials outlined in the previous subsection. The endpoints of overall median survival, overall survival at 6 months, 1-year local control, overall response rate, qol, neurologic function, and symptom control are reported when available. The number of qol instruments used in each study is also recorded.

### 3.4 QOL Instruments

A total of 24 different qol instruments, including performance scales, study-designed performance instruments, validated qol instruments, study-designed qol assessments, neurologic function scales, study-designed neurologic instruments, and ncf tests were used in the trials (Tables X–XIV). Six studies did not use any qol measures 20,43,60–62,78. The most commonly used instruments were the kps scale (*n* = 33) and various forms of neurologic function classification (*n* = 21). The number of qol instruments used in each study varied from 0 to 8, but most of the studies used 1 (*n* = 26, 43%) or 2 (*n* = 21, 34%) instruments. Of the 23 different instruments used, 8 (35%) assessed qol, 7 (30%) assessed ncf, 5 (22%) assessed performance status, and 3 (13%) assessed neurologic function.

Of the 8 qol instruments used, 2 were study-designed assessments 68,74. Kondziolka *et al.* 68 designed a 10-item survey to ask patients treated with wbrt and radiosurgery or with radiosurgery alone about their treatment perceptions, side effects (hair loss, fatigue, memory, mood or affect, intellectual concentration, employment), activity level, and overall satisfaction. This survey was used in a patient population in which 90% of the patients had a kps status of 90 or 100. After wbrt, the side effects reported were alopecia (88%); excess fatigue (85%); problems with short-term memory (72%), long-term memory (33%), and concentration (61%); and depression (54%). Also, patients more frequently reported short-term memory problems (*p* < 0.0001), long-term memory problems (*p* = 0.03), and concentration problems (*p* = 0.0007) when they had undergone both wbrt and radiosurgery as compared with radiosurgery alone. More patients considered radiosurgery a good treatment for them as compared with wbrt (76% vs. 56%, *p* = 0.25) 68

Sehlen *et al.* 74 developed the Current Situation in Personal Life questionnaire because previous trials had indicated that psychological and sociodemographic variables could influence survival in cancer patients 74. These authors assessed patients (kps > 70) who had undergone wbrt for primary central nervous system tumours or brain metastases; their instrument was designed to assess important sociodemographic variables and factors in the patients’ personal lives, such as marital status, number of children or people in the household, level of education, employment, family history of cancer, symptoms, relationships with family and friends, social life, hobbies, religion, and significant events. Interestingly, the results showed that “living with a spouse” had a statistically significant positive influence on survival (*p* = 0.033) 74.

Addeo *et al.* 55, Bezjak *et al.* 71, and Sehlen *et al.*^74^ used the Functional Assessment of Cancer Therapy—General scale (fact-g). This questionnaire is a validated instrument that evaluates the qol of cancer patients in 5 domains, including physical well-being (7 items), social or family well-being (7 items), relationship with the physician (2 items), emotional well-being (5 items), and functional well-being (7 items) 79. Sehlen and her colleagues showed that the overall fact-g score had a significant influence on survival (*p* = 0.003) 74.

The fact-g is often supplemented by site-specific questionnaires such as the fact–Brain subscale (fact– br) as used by Bezjak *et al.* 71 and Addeo *et al.* 55 The fact-br subscale contains 19 additional items pertaining to patients with brain metastases specifically, including symptoms, self-care, cognitive ability, and ease in usual activities 79. Bezjak *et al.* 71 found that, as compared with baseline, 8 of 23 patients showed improvement and 15 patients showed deterioration in assessed qol using the fact-g and fact-br questionnaires 1 month after palliative radiotherapy.

The full fact-br scale contains 53 questions (as compared with the subscale, with its 19 questions). Addeo *et al.*^55^ used the fact g and selected 26 items from the fact-br scale to assess qol in patients who underwent wbrt and temozolomide treatment. A significant improvement in qol was seen with the fact-g questionnaire (*p* < 0.0001). At baseline, 51% of patients reported, positively, that they were “quite a bit” or “very much” content with the quality of their life; 49% reported, negatively, that they were “not at all” or “a little bit” content with the quality of their life. Three months after treatment, 79% were content with their qol, and 21% were not content.

Gerrard *et al.* 72, Yaneva *et al.* 75, and Roos *et al.*^39^ used the European Organization for Research and Treatment of Cancer (eortc) Core Quality of Life Questionnaire (qlq-C30). This general questionnaire consists of 5 domains assessing functioning (physical, role, cognitive, emotional, and social), 1 domain assessing global qol, 3 domains assessing common symptoms (fatigue, pain, nausea or vomiting), 5 single items assessing other symptoms (dyspnea, insomnia, anorexia, constipation, and diarrhea), and 1 item assessing financial impact.

Yaneva *et al.* 75 evaluated the qol of patients with a kps greater than 70 before and after wbrt treatment. A significant improvement was evident after radiotherapy in all domains of functioning and in all symptoms with the exception of dyspnea, diarrhea, and financial difficulties. A significant improvement in health-related qol was also reported (*p* < 0.0001).

Gerrard *et al.* 72 and Roos *et al.* 39 used the supplementary Brain Cancer Module (bcm) in addition to the eortc qlq-C30. However, the bcm was designed for patients with primary brain tumour. It consists of 20 questions that assess side effects of treatment, outlook for the future, and common symptoms 80. Validation of this instrument in patients with brain metastases has not been reported.

When using the eortc qlq-C30 and bcm, Gerrard *et al.* 72 experienced difficulties with data collection and found that the questionnaires were lengthy and demanding, particularly for their poor-prognosis group. From the 18 patients analyzed in their first study, high levels of fatigue and drowsiness were seen throughout the study period (baseline to 8 weeks) and only 1 patient and 2 patients improved in qol at 2 weeks and 4 weeks respectively. Their second study, which also used the eortc qlc-30 and bcm 20, was terminated prematurely because of difficulties with data collection. Improvement in qol was not evident in any of the 6 patients accrued. Subsequently, in a third study, these authors simplified their qol assessment by asking only the global health score and global qol items of the questionnaire. Of 14 patients, 7 experienced transient improvements at some stage following wbrt.

Similarly, the randomized study of wbrt or control group post surgery or post radiosurgery by Roos *et al.* 39 was also terminated prematurely because of its slow accrual. As a result of the small sample size (*n* = 19), the investigators did not conduct a detailed qol analysis. They found that the differences in the global health scores and global qol scores between the two study arms were nonsignificant at 2 months (*p* = 0.94) and at 5 months (*p* = 0.50). The investigators concluded that their study did not indicate that wbrt caused deterioration in overall health or overall qol.

Chow *et al.* 17 used the Edmonton Symptom Assessment System (esas) in their study of patient-rated symptoms in patients with brain metastases treated with wbrt. The esas is a validated instrument designed for patients receiving palliative care. It evaluates 9 symptoms, including global pain, nausea, anxiety, depression, tiredness, drowsiness, sense of well-being, appetite, and shortness of breath. Each symptom is rated on a scale from 0 to 10, where 0 represents absence of the symptom and 10 represents the worst possible symptom. The esas has been shown to be a quick tool to use and to predominantly reflect the physical well-being of the patient 81. In the study by Chow and colleagues, 19%, 20%, and 15% of the patients died during the first, second, and third month following wbrt. The study population had statistically significant deterioration in the mean differences between their 1-year follow-up and baseline scores for fatigue (1.0 to 1.8), drowsiness (1.2 to 1.8), and appetite (2.2 to 2.4).

Mintz *et al.* 29 and Scott *et al.* 70 used the Spitzer Quality of Life index (Spitzer Q-L index). This validated instrument is composed of 5 domains: general activity, daily living, health, support, and outlook. Each domain is rated from 0 to 2 and each score is accompanied by verbal descriptions. For example, for the health domain, the patient could report either feeling well or “great” most of the time (score 2), lacking in energy or being not entirely “up to par” occasionally (score 1), or feeling very ill or “lousy,” weak and washed out for most of the week (score 0) 82.

Mintz *et al.* 29 conducted a controlled trial in which patients with a single brain metastasis were randomized to either wbrt and surgery or to wbrt alone. When comparing the two study arms, the mean qol scores were not significantly different at either of the study periods analyzed (1–3 months and 4–6 months).

Scott *et al.*^70^ assessed the qol of patients randomized to wbrt with efaproxiral or to wbrt alone. At the 6-month follow-up as compared with baseline, patients in the wbrt and efaproxiral arm had higher Spitzer Q-L scores than did the patients in the wbrt arm (*p* = 0.019). The authors also indicated that a score of 7 or better out of 10 before treatment was a significant predictor of overall survival. Patients with a score of 7 or better experienced a 48% reduction in death rate (*p* = 0.0079).

Regine *et al.* 77 used the Profile of Mood States–Short Form (poms-sf), a 30-item questionnaire organized into 6 mood scales: tension–anxiety, depression–dejection, anger–hostility, vigour–activity, fatigue–inertia, and confusion–bewilderment. The individual scales are combined to achieve an indicator of overall mood. A higher mood disturbance score indicates greater mood disturbance 77. Although compliance rates for completion of the poms-sf was high before treatment (95% or more), at treatment completion (84% or more), and at 1 month after treatment (70% or more), the results of the questionnaire were not reported because patient mood was not the primary objective of the study 77.

### 3.5 Performance Evaluation

The kps (discussed earlier) was the tool most commonly used to assess performance status in thirty-three studies. Results from Patchell *et al.* 34 are highlighted, because these authors used the kps as a measurement of qol when comparing patients with a single brain metastasis who had undergone either surgery and wbrt or wbrt alone. The length of time that kps scores remained at 70 or better was used as a determinant of qol. Patients in the surgery and wbrt arm maintained kps scores of 70 or better for much longer than did patients who received radiation alone (38 weeks vs. 8 weeks, *p* < 0.005). In a prospective study, Li *et al.* 38 compared the outcomes of 3 treatment arms in patients with a single brain metastasis and a kps score of 60 or better. An increase in kps score was seen in all 3 treatment arms: 88.9% (*n* = 16), 87.0% (*n* = 20), and 48.3% (*n* = 14) in patients who underwent radiosurgery in combination with wbrt, radiosurgery alone, and wbrt alone respectively. A greater improvement in kps was seen in patients treated with radiosurgery alone or with radiosurgery in combination with wbrt.

A study by Rosenman *et al.* 63 found that elective radiation could improve the qol of patients with small-cell lung carcinoma, although it did not increase the patients’ survival. All patients initially received a standard course of wbrt. After that course of treatment, 28 patients received elective radiation, and 24 patients received radiation only when brain metastases occurred (“therapeutic radiation”). A kps score above 60 was used by the investigators as a measure of qol. Patients in the electively radiated arm maintained a kps score greater than 60 for a mean time of 10 months as compared with a mean time of 6 months for patients in the therapeutically radiated arm.

The Eastern Cooperative Oncology Group (ecog) evaluation was used in eleven of the studies to determine performance status. Unlike the kps, which ranges from 0 to 100, the ecog is simpler. It ranges from 0 to 4, where 0 stands for “normal activity” and 4 means “unable to get out of bed.” Roos *et al.* 39 used ecog as a part of their qol assessment (baseline vs. first follow-up) when comparing patients randomized to wbrt or to observation after surgery or radiosurgery. No significant difference was found between the two study arms (*p* = 0.80).

Five of the studies used the General Performance Status (gps), which ranges from 1, which means “normal,” to 5, which means “100% bedridden.” Also, two of the studies used the Barthel index of activity of daily living, which is a validated measure for patients with neurologic disability. Its questions focus on physical performance in 10 areas: feeding, transfers from bed to chair and back, grooming, toilet use, bathing, mobility, climbing stairs, dressing, stool control, and bladder control.

Finally, four of the studies designed their own scales to evaluate performance status 22,23,40,50. For example, Horton *et al.* 40 measured performance status using a scale from 0 (“normal performance”) to 4 (“completely bedridden”). Kurtz *et al.* 50 measured performance status on a scale from 0 to 100, where scores from 70 to 100 indicated ambulatory patients and scores under 70 indicated non-ambulatory patients. Harwood *et al.* 22 classified the functional status of their patients by level i, ii, iii, and iv, where level i meant that the patient was “intellectually and physically able to work with neurological abnormalities minor or absent” and level iv meant the patient had “profound neurologic disability.” Noordijk and colleagues assessed the general well-being of the patients by designing a functionally independent survival tool 23. Patients were considered to be functionally independent as long as their score on the ecog scale was 1 or lower (symptomatic, but almost completely independent) and their score on a version of a neurologic function classification was 1 or lower (patient can perform normal activities with minimal difficulties).

### 3.6 Evaluation of Neurologic Function and Symptoms

In 23 studies (Table XIII), a measure of the neurologic function and symptoms of the patients was reported. Various versions of a neurologic functional classification or scale was used in 20 reports. Bezjak *et al.* 71 modelled an assessment tool after symptom items included in the fact-br and the bcm 20. This patient-rated assessment tool consisted of 16 items specific to patients with brain metastases. Symptoms were subdivided into raised intracranial pressure (3 items), effects associated with steroid use (4 items), possible subacute side effects (4 items), and effects associated with brain metastases (5 items). Robinet *et al.* 7 used the order classification to record the neurologic status of the patients.

### 3.7 Neurocognitive Function

Li *et al.* 76, Murray *et al.* 27, Regine *et al.* 77, Roos *et al.* 39, Scott *et al.* 70, and Sehlen *et al.* 74 assessed ncf in their studies, five of which included the Mini Mental Status Examination (mmse) as an instrument 27,39,70,74,77 (Table XIV). The mmse is a validated and easily administered tool consisting of 11 items designed to test cognitive function. It includes tests of the patient’s knowledge of orientation (1 item); memory (2 items); immediate recall (1 item); attention, concentration, and calculation (1 item); and aphasia and apraxia (4 items) 27 Roos *et al.*^39^ compared the mmse scores of patients with a single brain metastasis randomized to wbrt or to observation post surgery or post radiosurgery. Although the study was terminated prematurely because of slow accrual, no significant difference was found between the two study arms at the 12-month follow-up (*p* = 0.50).

The Hopkins Verbal Learning Test, which was used by Li *et al.* 76 and Regine *et al.* 77 is a memory test instrument and includes items for short- and long-term recall and word recognition 83. The Controlled Oral Word Association test used by Li *et al.* 76 and Regine *et al.* 77 assesses language and executive function skills where the patient’s task is to produce, in 1 minute, as many words as possible beginning with a specific letter. Additionally, trials by Li *et al.* 76 and Regine *et al.* 77 used the trail-making test designed to test visual motor speed and executive function 83.

Regine *et al.* 77 included the Ruff 2 and 7 Test as a component of their ncf test battery to assess neglect, attention, and concentration. Li *et al.* 76 assessed motor speed, visual–motor coordination, and single-hand dexterity with the Grooved Pegboard Test. Sehlen *et al.* 74 used a modified abbreviated version of the Mini Mental State Inventory to evaluate mental capacity.

## 4. DISCUSSION

In recent years, qol has become an increasingly important outcome in cancer trials. To date, fourteen trials on brain metastases that included an evaluation of the study population’s qol have been published. Three of the trials used the fact-g and fact-br instruments, three used the eortc qlq-C30 and bcm 20 instruments, two designed qol instruments specifically for the trial, one used the esas instrument, two used the Spitzer Q-L index, and three used the kps as a tool to evaluate qol. Our findings suggest that, although numerous qol questionnaires exist, no standard questionnaire is currently used to assess qol in patients with brain metastases. Currently, the use of these different questionnaires does not allow for a comparison of qol across trials. A standard tool would be beneficial for comparisons across trials and for performing meta-analyses.

Our literature review shows that certain parameters of qol deteriorate after wbrt 17,71,72. Chow *et al.* 17 concluded that the esas domains of fatigue, drowsiness, and appetite worsened after wbrt in their patients (baseline median kps: 60; range: 20–90). In the study by Gerrard *et al.* 72, 10 of the 38 patients (26%; 95% confidence interval: 13%–43%) improved in at least one of the following parameters during the study period: qol score, Barthel index of activity of daily living, or kps 8 weeks after wbrt. However, 14 of 15 patients had deterioration in at least one of these parameters. Using the fact-br questionnaire, Bezjak *et al.*^71^ also found deterioration in qol from baseline to 1 month, but the difference was not statistically significant (*p* = 0.13). These findings have led authors to question whether patients with poor prognosis benefit from radiotherapy in terms of effect on qol and symptom experiences 17,72.

For patients with a better prognosis, the results of Addeo *et al.* 55, Yaneva *et al.* 75, and Scott *et al.* 70 showed that certain parameters of qol significantly improved after wbrt Addeo *et al.*^55^ used the fact-g and 26 of the fact-br scale items to assess qol in patients who underwent wbrt and temozolomide treatment. A significant improvement in qol was seen (*p* < 0.0001). Three months after treatment, 79% were content with their quality of life, and 21% were discontent (compared with 51% positive respondents and 49% negative respondents at baseline).

Using a recursive partitioning analysis (rpa) based on the kps, the Radiation Therapy Oncology group established three prognostic classes for patients with brain metastases according to tumour, primary tumour status, presence of extracranial metastases, and age. Class i included patients with a kps of 70 or better, age below 65 years, no extracranial metastases, and a controlled primary tumour; these patients had a median survival of 7.1 months. In comparison, patients with a kps below 70 are class iii with a median survival of 2.3 months. All other patients belong to class ii, with a median survival of 4.2 months.

Addeo *et al.* 55 included a high number of patients in the rpa classes i (*n* = 21, 36%) and ii (*n* = 22, 37%). That patient population differed greatly from the population included in the study by Bezjak *et al.* 71, where 3, 31, and 41 patients were in rpa classes i, ii, and iii respectively.

Yaneva *et al* 75 used the eortc qlq-C30 in a patient population who underwent wbrt. Significant improvements in functional indicators, symptoms, and health-related qol were found after wbrt. Those results differ from the findings of Gerrard *et al.* 72, who also used the eortc qlq-C30 questionnaire; however, the population in their study satisfied at least two of the following criteria: kps below 70, more than 60 years of age, or a primary tumour site other than breast. In comparison, Yaneva *et al.* 75 selected patients who had kps scores above 70.

Scott *et al.* 70 randomized patients to wbrt with efaproxiral or to wbrt alone, using the Spitzer Q-L index as a measurement of qol. At the 6-month follow-up, patients who had received wbrt and efaproxiral had higher qol scores than did the patients who had received wbrt alone (*p* = 0.019). This study population also included patients with a better prognosis (only rpa class i and ii patients were included). Of the study population, 58% percent had a kps score of 90–100, and 42% had a kps score of 70–80.

One study found that certain parameters of qol did not deteriorate or improve after wbrt. Roos*et al.* 39 randomized patients to wbrt or to no additional treatment post surgery or post radiotherapy. The eortc global health scores and global qol scores were not significant between the study arms at 2 months (*p* = 0.94) and at 5 months (*p* = 0.50). These patients also had a fairly good prognosis: solitary brain metastasis and 14, 4, and 1 of 19 patients in rpa classes i, ii, and iii respectively. Although no improvement in qol was evident, the results also did not indicate that qol deteriorated after patients received wbrt. Poor accrual and low statistical power likely contributed to this outcome.

The present review found that few wbrt studies included a measure of qol as a primary endpoint. A possible explanation is the difficulty in collecting data in a population of patients whose life expectancy is short. Patients with short survival and deterioration of health often contribute to high attrition rates in brain metastases qol studies 17,72 For example, Bezjak *et al.*^71^ found that only 19% of patients had symptomatic improvement and that 55% had either progressed in their illness or had died at 1 month. Consequently, the drop-out bias affecting research studies must be kept in mind: patients included in the results are those able to complete follow-up assessments and are thus likely have a better prognosis than are the patients lost to follow-up 11,71. Scott *et al.* 70 found that the Spitzer Q-L index was a better predictor of survival than the kps was, and they suggested the use of this qol instrument in predicting survival and assessing patient status. Sehlen and colleagues found that the overall fact-g score had a statistically significant correlation with survival (*p* = 0.003). Although data collection is a challenge in this study population, the results of Sehlen *et al.* 74 suggest that qol is a worthwhile endpoint to include in future brain metastases trials and that it could possibly distinguish patients with a longer expected survival.

The studies identified in this review used 55 different performance status assessment tools and 23 different neurologic function instruments. However, these instruments were primarily used to categorize the patients into prognostic groups, to describe the study population, or to act as exclusion criteria. The study by Patchell *et al.* 34 was an exception: the authors used the kps to evaluate the qol of patients before and after treatment. They determined qol by the length of time the kps remained at 70 or higher. Their results showed that the kps scores of patients in the combined radiotherapy and surgery arm were maintained for a much longer period than were the scores of patients who had undergone radiotherapy alone (38 weeks vs. 8 weeks, *p* < 0.005) 34. Similarly, Li *et al.* 38 compared kps scores from the day of treatment with scores from the first follow-up visit to determine if different treatments had an effect on the qol of lung cancer patients with a single brain metastasis. Improvements of 88.9% (*n* = 16) and 87.0% (*n* = 20) respectively were seen in the kps scores of patients who underwent radiosurgery in combination with wbrt and radiosurgery alone. In comparison, an improvement of 48.3% (*n* = 14) was seen in patients who underwent wbrt alone. A study by Rosenman *et al.*^63^ investigated whether qol improved with elective radiation after a standard course of wbrt in 28 patients (compared with 24 patients who received radiation therapeutically). These authors defined qol as the length of time a patient’s kps score remained above 60. Patients in the electively radiated arm maintained a kps score above 60 for significantly longer than did the patients in the therapeutically radiated arm (10 months vs. 6 months).

The ncf is clearly an important concern for brain metastases patients. Although the mmse was the most frequently used measure of ncf in the studies, it is less sensitive to mild neurocognitive impairment and may not identify subtle improvements 68,83. In addition, the mmse has not been as thoroughly evaluated in patients with brain metastases as compared with patients with primary brain tumours 83. Hence, studies have designed ncf test batteries to thoroughly evaluate the ncf of study patients 77,83. Li *et al.* 76 investigated the ncf of patients who had been treated with a radiosensitizer (gadolinium) and wbrt. Patients were classified as “good” or “poor” responders depending on whether their tumour reduction at 2 months was above or below the population median reduction of 45%. Their results showed that the “good” responders survived significantly longer than did the “poor” responders. Time to ncf deterioration was compared in the “good” and “poor” responders, and results indicated that patients with volume regression after radiation had a longer delay before ncf deterioration. The authors concluded that ncf and qol correlated in their study population and that efforts to prevent the worsening of ncf could help maintain qol 76.

## 5. CONCLUSIONS

Quality of life is an important outcome in the treatment of patients diagnosed with brain metastases. However, few clinical trials have focused on qol as a primary outcome. Common outcomes measured are survival, response to treatment, symptomatic relief, toxicity, and duration of independent function. The present review finds that various management methods for brain metastases have been explored, and yet median survival in this patient population has not improved significantly. Thus, less-morbid treatment options that preserve or improve qol in these patients are important.

Our literature review found that a number of qol instruments have been used to evaluate patients with brain metastases. Additional assessment tools, including performance status tools, neurologic function assessments, and ncf tests were also used in many clinical trials to evaluate the well-being of patients. Some studies have shown that certain parameters of qol deteriorate after wbrt in patients with poorer prognosis, but other studies have shown that qol in patients with better prognosis improve after wbrt. Although a number of validated qol questionnaires specific to the concerns of metastatic brain cancer patients have been developed, no standard questionnaire has currently been established for this patient population, making comparisons of qol across trials difficult. Our findings emphasize the importance of including qol as an endpoint in future clinical trials so as to better understand the role of qol, especially for improving treatment in patients with brain metastases.

## Figures and Tables

**TABLE I:** Studies involving patients with a single brain metastasis

Reference	Study arms	Patients (n)	Survival			Tools		qol instruments (n)
			Median (months)	Overall at 6 mo. [n (%)]	qol assessment	Neurologic function or symptom control	Other assessment	
Auchter *et al.*, 1996 36	Radiosurgery + wbrt (range: 25–40 Gy)	122 a	14	(53 at 1 year)	kps		Duration of functional independence Cause of death	1
Epstein *et al.*, 1993 35	32 Gy in 20 fractions bid + boost:				kps	Neurologic function classification		2
	48 Gy	30	4.9					
	54.4 Gy	53	5.4					
	64 Gy	44	7.2					
	70.4 Gy	26	8.2					
Jyothirmayi *et al.*, 2001 37	Radiosurgery (at diagnosis)	45	10		kps		Overall response rate	1
	Radiosurgery + wbrt^b^ (at diagnosis)	22	8				Toxicity	
	Radiosurgery (at recurrence)	29	7					
Li *et al.*, 2000 38	35–45 Gy/18–25 fractions	29	5.7 (*p<*0.0001)		kps		Intracranial progression-free duration Overall response rate	1
	Radiosurgery	23	9.3				Cause of death	
	Radiosurgery + 30–45 Gy/15–25 fractions	18	10.6					
Mintz *et al.*, 1996 29	30 Gy/10 fractions + surgery	41	5.6 *p=* ( ns)	19 (46)	Spitzer’s QL index		Cause of death	2
	30 Gy/10 fractions	43	6.3	23 (53)	kps			
Noordijk *et al.*, 1994 23	40 Gy/20 fractions bid + surgery	32	10	21 (66)	ecog	Neurologic function classification	Cause of death	3
	40 Gy/20 fractions bid	31	6	16 (52)	fis			
Patchell *et al.*, 1990 34	36 Gy/12 fractions + surgery	25	9.2 (*p<*0.01)	17 (68)	kps		Duration of functional independence Cause of death	1
	36 Gy/12 fractions	23	3.5	5 (22)				
Patchell *et al.*, 1998 19	Surgery + 54 Gy/28 fractions	49	48 weeks (*p=*0.39)		kps		Cause of death	1
	Surgery	46	43 weeks					
Roos *et al.*, 2006 39	36 Gy/18 fractions	10	9.2 (*p=* NS)		ecog		mmse	3
	Control group (1 in each arm, surgery; 17 patients had radiosurgery)	9	6.2		eortc qlq-C30 and bcm 20			

a 5 Patients declined wbrt, but were included in the study.

b 76% of the study population received 20 Gy/ 2 fr.

c abstract only.

qol = quality of life; wbrt = whole-brain radiation therapy; kps = Karnofsky performance status; bid = twice daily; ecog = Eastern Cooperative Oncology Group; ns = nonsignificant; fis = Functionally Independent Survival; eortc qlq-30 = European Organization for Research and Treatment of Cancer Core Quality of Life Questionnaire; eortc bcm 20 = European Organization for Research and Treatment of Cancer Brain Cancer Module.

**TABLE II:** Studies of whole-brain altered-fractionation radiotherapy for brain metastases

Reference	Study arms	Patients (n)	Survival		Tools		qol
			Median (range)	Overall at 6 mo. [n (%)]	qol assessment	Neurologic function or symptom control	instruments (n)
Bach *et al.,* 1996 51	50.4 Gy/28 fractions, 5 fractions/week	44	160 days (74–2021 days) a		ecog		1
	22 Gy/4 fractions	57	88 days				
	sclc patients post-chemotherapy	*(p*<0.00001)	(20–948 days),				
Borgelt *et al.,*	Study 1:						
1980^10^	30 Gy/10 fractions	233	4.2 months		gps	Neurologic function classification	2
	30 Gy/15 fractions	217	(3.7–4.6 months)				
	40 Gy/15 fractions	233	*(p*>0.05)				
	40 Gy/20 fractions	227					
	Study 2:						
	20 Gy/5 fractions	447	3.5 months				
	30 Gy/10 fractions	228	(3.2–3.5 months)				
	40 Gy/15 fractions	227	*(p*>0.05)				
Borgelt *et al.,* 1981 44	Study 1:				gps	Neurologic function classification	2
	10 Gy/1 fractions	26	3.5 months				
	30–40 Gy/10–20 fractions	112	4.8 months *(p*>0.05)				
	Study 2:						
	12 Gy/2 fractions	33	3.0 months				
	20 Gy/5 fractions	31	2.8 months *(p*>0.05)				
Chatani *et al.,* 1994 45 (abstract)	Normal lactate dehydrogenase (ldh):						0
	30 Gy/10 fractions	46	5.4 months	19 (41)			
	50 Gy/20 fractions	46	4.8 months *(p*=0.841)	22 (48)			
	High ldh						
	30 Gy/10 fractions	35	3.4 months	7 (20)			
	50 Gy/20 fractions	35	2.4 months *(p*=0.943)	7 (20)			
Gelber *et al.,* 1981 46	20 Gy/5 fractions	Breast patients: 160	33 weeks		gps	Neurologic function classification	2
	30 Gy/10 fractions	Lung patients: 556	20 weeks				
	30 Gy/15 fractions	Other patients: 213	20 weeks				
	40 Gy/15 fractions						
	40 Gy/20 fractions						
Haie–Meder *et al.,* 1993 28	1 course:						
	18 Gy/3 fractions/3 days	110	4.2 months	53 (48)	kps		1
	2 courses:		*(p*>0.05)				
	18 Gy/3 fractions/3 days followed 1 month later by another 18 Gy/3 fractions/3 days, or 18 Gy/3 fractions/3 days followed 1 month later by another 25 Gy/10 fractions/14 days	106	5.3 months	41 (38)			
Harwood *et al.,* 1977 22	10 Gy/1 fractions	51	4.4 months *(p*=0.082)	14 (27)	Functional status	Neurologic function classification	2
	30 Gy/10 fractions	50	4.0 months	20 (40)			
Kurtz *et al.,* 1981 50	30 Gy/10 fractions	130	18 weeks *(p* = ns)		Performance status	Neurologic function classification	2
	50 Gy/20 fractions	125	17 weeks				
Murray *et al.,* 1997 6	54.4 Gy/34 fractions bid (over 17 days)	216	4.5 months *(p*=0.52)	84 (39)	kps	Neurologic function classification	2
	30 Gy/10 fractions (over 10 days)	213	4.5 months	88 (41)			
Nieder *et al.,* 1997 47	30 Gy/12 fractions bid + surgery	11	3.3 months		kps		1
	30 Gy/12 fractions bid	36	2.0 months				
	50.4 Gy/28 fractions bid	15	2.0 months				
	30 Gy/10 fractions (historical group)	246	2.5 months				
	30 Gy/10 fractions + surgery (historical group)	37	7.3 months				
Portaluri *et al.,* 2004 48	50 Gy/25 fractions	26	4 months (mean survival)	6 (21)		Neurologic function classification	1
	30 Gy/10 fractions	48	5 months	18 (36)			
	20 Gy/5 fractions	42	5 months	9 (21)			
	9 Unusual fractionation treatments b						
Priestman *et al.,* 1996 49	30 Gy/10 fractions	263	2.8 months *(p*=0.04)	46 (17)	ECOG	Neurologic function classification	2
	12 Gy/2 fractions	270	2.5 months	66 (25)			

a Measured from the diagnosis of brain metastases.

b Exact dose and fractionation schedule not described.

qol = quality of life; sclc = small-cell lung cancer; ecog = Eastern Cooperative Oncology Group assessment; gps = General Performance Status; kps = Karnofsky performance status; ns = nonsignificant; bid = twice daily.

**TABLE III:** Multiple brain metastases: studies of whole-brain radiotherapy (wbrt) with radiosensitizers compared with wbrt alone

Reference	Study arms	Patients (n)	Median survival	Overall response rate (cr+pr %)	qol assessment	Tools Neurologic function or symptom control	Other assessment	qol instruments (n)
DeAngelis *et al.,* 1989 9	30 Gy/10 fractions + lonidamine 30 Gy/10 fractions	19	4.0 months *(p*= ns)	37% (11.5 patients)	kps		Cause of death Toxicity	1
		20	5.4 months	55% (15 patients)				
Eyre *et al.,* 1984 24	30 Gy/10 fractions + metronidazole 30 Gy/10 fractions	57	2.8 months *(p*= ns)	27% (15 patients)		Neurologic function classification	Cause of death Toxicity	1
		54	3.2 months	24% (13 patients)				
Johnson *et al.,* 1998 52	wbrt + pentoxifylline	14	33 days	14% (2 patients)	ecog	Neurologic function classification		2
Kocher *et al.,* 2005 53	36 Gy/12 fractions + topotecan	47	5.1 months	58% (15 patients)	kps		Cause of death Toxicity	1
Phillips *et al.,* 1995 21	37.5 Gy/15 fractions + bromodeoxyuridine 37.5 Gy/15 fractions	34	4.3 months *(p*= ns)	63% of 22 patients evaluable for response (14 patients)	kps	Neurologic function classification	Toxicity	2
		36	6.12 months	50% of 24 patients evaluable for response (12 patients)				
Rhomberg *et al.,* 2005 25	30 Gy/30 fractions + boost series of 2 Gy/dose, median of 43 Gy + razoxane 30 Gy/30 fractions + boost series of 2 Gy/dose, median dose of 35 Gy	8	5 months *(p*= ns)	62% (5 patients)	kps	Score Index for Stereotactic Radiosurgery (sir) for Brain Metastasis	Toxicity	1
		11	2.2 months	27% (3 patients)				
Stea *et al.,* 2006 54	30 Gy/10 fractions + efaproxiral 30 Gy/10 fractions	265 a		74% (27.9 patients) c				
		250 b		50% (20 patients) b	kps			1

a 18 Patients had radiosurgery and 3 patients had surgical resection after randomization.

b 13 Patients had radiosurgery and 9 patients had surgical resection after randomization.

c Response rate determined 3 months after treatment.

cr = complete response; pr = partial response; qol = quality of life; ns = nonsignificant; kps = Karnofsky performance status; ecog = Eastern Cooperative Oncology Group assessment.

**TABLE IV:** Multiple brain metastases: studies assessing the efficacy of whole-brain radiotherapy (wbrt) and chemotherapy

Reference	Study criteria	Study arms	Patients (n)	Median survival	Overall response rate (cr+pr)	qol assessment	Tools Neurologic function or symptom control	Other assessment	qol instruments (n)
Addeo *et al.,* 2007^55^	Metastatic cancer to the brain	30 Gy/10 fractions + temozolomide	59	13 months	44%	kps fact-g fact-br		Toxicity	2
Antonadou *et al.,* 2002^56^	Metastatic cancer to the brain	30 Gy/10 fractions + temozolomide	Total of 134 patients randomized	8.3 months	53.4%	kps			1
		30 Gy/10 fractions		6.3 months (*p*=0.179)	33.3% (*p*=0.039)				
Guerrieri *et al.,* 2003^57^	Metastatic nsclc to the brain	20 Gy/5 fractions + carboplatin	21	3.7 months	29%	ecog	Neurologic function classification	Toxicity	2
		20 Gy/5 fractions	21	4.4 months (*p*=0.64)	10% (*p*=0.24)				
Hidalgo *et al.,* 1987^58^	Metastatic cancer to the brain	50 Gy/25 fractions + *cis-*platinum	13	n/a	92.3% (12 patients)	kps		Toxicity	1
Postmus *et al.,* 2000^8^	Metastatic sclc to the brain	30 Gy/10 fractions + teniposide	60	3.5 months	57%	ecog	Neurologic function classification	Toxicity Duration till progression	2
		Teniposide	60	3.2 months (*p*=0.087)	22% (*p*<0.001)				
Robinet *et al.,* 2001^7^	Metastatic nsclc to the brain	Delayed 30 Gy/10 fractions + cisplatin and vinorelbine	76	6.0 months	33%	ecog	Order classification	Toxicity	2
		Early 30 Gy/10 fractions + cisplatin and vinorelbine	73	5.3 months (*p*=0.83)	27% (*p*=0.12) Intracranial response rate				
Ushio *et al.,* 1991^60^	Metastatic lung cancer to the brain	40 Gy total (1.5–2 Gy per dose)	31	27 weeks	36%				0
		wbrt + chloroethylnitrosoureas	36	29 weeks	69%				
		wbrt + chloroethylnitrosoureas + tegafur	33	24 weeks (*p*=ns)	74% (*p*<0.05) Brain metastases regression				
Verger *et al.,* 2004^59^	Metastatic cancer to the brain	30 Gy/10 fractions + temozolomide	41	4.5 months	32%	kps		Toxicity	2
		30 Gy/10 fractions	41	3.1 months (*p*=ns)	32% (*p*=ns at 30 days)	Barthel index of activities of daily living			

cr = complete response; pr = partial response; qol = quality of life; fact-g = Functional Assessment of Cancer Therapy—General scale; fact-br = Functional Assessment of Cancer Therapy—Brain subscale; kps = Karnofsky performance status; nsclc = non-small-cell lung cancer; ecog = Eastern Cooperative Oncology Group assessment; sclc = small-cell lung cancer; ns = nonsignificant.

**TABLE V:** Multiple brain metastases: studies assessing the efficacy of whole-brain radiotherapy (wbrt) with or without radiosurgery

Reference	Study criteria	Study arms	Patients (n)	Median survival	1-Year local control	qol assessment	Tools Neurologic function or symptom control	Other assessment	qol instruments (n)
Andrews *et al.,* 2004 18	1–3 Brain metastases	37.5 Gy/15 fractions + radiosurgery	164	6.5 months (*p*=0.1356)	82% (*p*=0.01)	kps		Cause of death Toxicity	1
		37.5 Gy/15 fractions	167	5.7 months	71%				
Jawahar *et al.,* 2002 66	Brain metastases	30 Gy/10 fractions	86	5 months (*p*=0.0016)		kps		Cause of death	1
		Gamma knife radiosurgery	48	12 months					
Kondziolka *et al.,* 1999 20	2–4 Brain metastases	30 Gy/12 fractions + Gamma Knife radiosurgery	13	11.5 months (*p*=0.22)	92% (*p*=0.01)				0
		30 Gy/12 fractions	14	7.5 months (*p*=ns)	0%				
Pirzkall *et al.,* 1998 67	1–3 Brain metastases	30–50 Gy total, median dose of 15 Gy + radiosurgery	78	5.5 months (entire study population)	92% (*p*=0.13)	kps		Cause of death	1
		Radiosurgery	158		89%				
Sneed *et al.,* 1999 69	Brain metastases	Radiosurgery + wbrt	43	11.1 months (*p*=0.80)	69%	kps		Cause of death	1
		Radiosurgery	62	11.3 months	28%				

qol = quality of life; kps = Karnofsky performance status; ns = nonsignificant.

**TABLE VI:** Multiple brain metastases: studies assessing the efficacy of re-irradiation (ri)

Reference	Study arms	Population sample	Overall median survival following ri	qol Assessment	Tools Neurologic function or symptom control	Other assessment	qol Instruments (n)
Abdel–Wahab *et al.,* 1997 65	Initial course:	15	3.2 months	kps		Response to treatment	1
	range 30–55 Gy, 1.5 Gy/fractions bid						
	Whole-brain RI:						
	median 30 Gy						
Hazuka *et al.,* 1988 61	Initial course:	37 whole-brain ri	8 weeks			Response to treatment	0
	median 30 Gy	7 partial-brain ri					
	Whole- or partial-brain ri:						
	median 25 Gy, 3.0 Gy/fractions						
Kurup *et al.,* 1980 62	Initial course:	56	3.5 months			Response to treatment	0
	18 Gy/3 fractions,						
	20 Gy/5 fractions,						
	30 Gy/10 fractions						
	Whole-brain ri:						
	Most patients received a single 5-Gy dose or 46 Gy/20 fractions (5 Gy/week)						
Rosenman *et al.,* 1982 63	Initial course:		Difference in survival:				
	30 Gy/10 fractions			kps		Response to treatment	1
	– Elective whole-brain ri	24	*p*=ns				
	– Therapeutic whole-brain ri^a^	28					
Shehata *et al.,* 1974 64	60% Whole-brain ri (single 10-Gy dose)	50	150 days	Neurologic function classification		Response to treatment	1

a Defined by authors, because patients were re-irradiated when brain metastases occurred.

qol = quality of life; bid = twice daily; kps = Karnofsky performance status; ns = nonsignificant.

**TABLE VII:** Multiple brain metastases: studies focused on quality of life (qol), neurologic function, and neurocognitive function (ncf)

Reference	Study arms	Patients (n)	Median survival	qol assessment	Tools Neurologic function or symptom control	Other assessment	qol instruments (n)
Bezjak *et al.,* 2002 71	20 Gy/5 fractions	75	86 days	ecog	Neurologic symptom checklist modelled after the fact-br and eortc bcm 20 Neurologic function classification	Analgesic measurement	5
				fact-g		mmse	
				fact-br			
Chow *et al.,* 2005 17	20 Gy/5 fractions (*n*=138)	170	8 weeks	kps			2
	30 Gy/10 fractions (*n*=7)			esas			
	Other dose fractionations (*n*=25)						
Gerrard *et al.,* 2003 72	First study:			kps, eortc qlq-30, and bcm 20			4
	12 Gy/2 fractions	18	6 weeks				
	Second study:			kps, Barthel index of activity of daily living, eortc qlq-30, and bcm 20	Neurologic function classification	Time to progression	
	20 Gy/5 fractions	6	8 weeks			Analgesic measurement	
						Side effects of treatment	
	Third study:			kps, Barthel index of activity of daily living, questions 29 and 30 of qlc-C30		Analgesic measurement	
	12 Gy/2 fractions or 20 Gy/5 fractions	14	10 weeks			Side effects of steroid and treatment	
Kondziolka *et al.,* 2005 68	30 Gy/12 fractions or 10 fractions and Gamma Knife radiosurgery a	72		kps			2
	Gamma Knife radiosurgery a	32		Study-designed questionnaire			
Li *et al.,* 2007 76	Motexafin gadolinium and 30Gy/10 fractions	208	Good responders:	kps		ncf test battery b:	5
			300±26 days c			hvlt, Trailmaking A and B, Grooved Pegboard, cowa	
			Poor responders:				
			240±19 days				
Lock *et al.,* 2004 73	wbrt—	275	5.3 months	ecog			1
	most frequent dose fractionation:						
	20 Gy/5 fractions (6% of patients received 30 Gy/10 fractions; 6% received other schedules)						
Murray *et al.,* 1999 27	30 Gy/10 fractions	182	4.2 months	kps		mmse	2
Regine *et al.,* 2004 77	37.5 Gy/15 fractions (2.5 Gy/fraction)	55		ecog	Neurologic function classification	ncf test battery b:	8
				poms-sf		mmse, hvlt, cowa, Ruff 2 and 7 and Trailmaking A and B	
Scott *et al.,* 2007 70	30 Gy/10 fractions + efaproxiral	57	9.0 months	kps	Neurologic function classification	mmse	4
	30 Gy/10 fractions	49	4.5 months *(p*=0.004)	Spitzer Q-L index			
Sehlen *et al.,* 2003 74	cns primary and radiotherapy	24	26.4 months d	kps, fact-g		mmsi (modified abbreviated version)	4
	Brain metastases and radiotherapy	33	28.3 months *(p*=ns)	Current Situation in Personal Life questionnaire			
Yaneva *et al.,* 2006 75	30 Gy/10 fractions or 15 fractions	65	6.6 months e	kps			2
			9.8 months f	eortc qlq-C30			

a 2–4 Brain metastases.

b Authors believed ncf and qol correlated in this population.

c Good responders showed tumour shrinkage above the population median; poor responders showed tumour shrinkage below the population median.

d Survival length is unexpectedly long (attributable to the patient sample, which contains patients with anaplastic astrocytomas (34.4%) and brain tumours of a different histologic origin (12.5%).

e Lung cancer patients.

f Breast cancer patients.

qol = quality of life; ecog = Eastern Cooperative Oncology Group Assessment; fact-g = Functional Assessment of Cancer Therapy—General scale; fact-br = Functional Assessment of Cancer Therapy—Brain subscale; eortc bcm 20 = European Organization for Research and Treatment of Cancer Brain Cancer Module; mmse = Mini Mental Status Examination; kps = Karnofsky performance status; esas = Edmonton Symptom Assessment Scale; eortc qlq-30 = European Organization for Research and Treatment of Cancer Core Quality of Life Questionnaire; hvlt = Hopkins Verbal Learning Test; cowa = Controlled Oral Word Association; wbrt = whole-brain radiotherapy; poms-sf = Profile of Mood States–Short Form; mmsi = Mini Mental State Inventory; ns = nonsignificant.

**TABLE VIII:** Studies focused on managing brain metastases through corticosteroids

Reference	Study arms	Patients (n)	Median survival	Overall response rate (cr+pr)	qol assessment	Tools Neurologic function or symptom control	Other assessment	qol instruments (n)
Horton *et al.,* 1971 40	Oral prednisone and wbrt	48 (total)	14 weeks		Performance status		Toxicity	1
	Oral prednisone		10 weeks				Incidence of remissions	
Wolfson *et al.,* 1994 41	All patients:		4 months (total population)	3 cr	gps	Neurologic function classification		2
	dexamethasone every 6 hours for 48 hours			1 pr				
				8 no response				
	Arm 1:	7						
	4 mg every 6 hours dexamethasone with 30 Gy/10 fractions							
	Arm 2:	5						
	30 Gy/10 fractions							

cr = complete response; pr = partial response; qol = quality of life; wbrt = whole-brain radiotherapy; gps = General Performance Status.

**TABLE IX:** Multiple brain metastases: studies assessing the efficacy of multiple treatments

References	Study arms	Patients (n)	Median survival	qol assessment	Tools Neurologic function or symptom control	qol instruments (n)
Chang *et al.,* 1992 42	nsclc patients			gps	Neurologic function classification	2
	Surgery (4 patients received 30 Gy/15 fractions)	9	9 months			
	Chemotherapy (4 patients received 30 Gy/15 fractions)	10	10 months			
	30 Gy/15 fractions	12	7 months a			
	Supportive care (2 patients received a ventriculoperitoneal shunt)	19	2 months			
Routh *et al.,* 1994 43	wbrt	223	2.5 months			0
	Cisplatin, etoposide + wbrt	5	(entire study population, from start of wbrt)			
	wbrt + wbri	16				
	wbrt + surgery	32				

a Difference between the three treatment modalities was nonsignificant.

qol = quality of life; nsclc = non-small-cell lung cancer; gps = General Performance Status; wbrt = whole-brain radiotherapy; wbri = whole-brain re-irradiation.

**TABLE X:** Frequency of instruments used in clinical trials measuring quality of life (qol) in patients with brain metastases

Instrument	Frequency
Karnofsky performance status	33
Neurologic function classification	21
ecog (World Health Organization) performance scores	11
General Performance Status	5
Mini Mental Status Examination	5
Study-designed performance instrument 22,23,40,50	4
Barthel index of activity of daily living	2
Controlled Oral Word Association test	2
Hopkins Verbal Learning Test	2
Spitzer quality of life index	2
Study-designed qol assessment 68,74	2
Trailmaking A and B	2
Edmonton Symptom Assessment Scale	1
eortc Core Quality of Life Questionnaire	1
with Brain Cancer Module	2
Functional Assessment of Cancer Therapy–General scale	1
With Brain subscale	2
Grooved Pegboard	1
Mini Mental State Inventory (modified abbreviated version)	1
Order classification	1
Profile of Mood States–Short Form	1
Ruff 2 and 7	1
Study-designed neurologic instrument 71	1

ecog = Eastern Cooperative Oncology Group; eortc = European Organization for Research and Treatment of Cancer.

**TABLE XI:** Frequency of instruments used in assessing quality of life (qol) in clinical trials

Instrument	Frequency
Spitzer quality of life index	2
Study-designed qol assessment 68,74	2
Edmonton Symptom Assessment Scale	1
eortc Core Quality of Life Questionnaire	1
with Brain Cancer Module	2
Functional Assessment of Cancer Therapy–General scale	1
with Brain subscale	2
Profile of Mood States–Short Form	1

eortc = European Organization for Research and Treatment of Cancer.

**TABLE XII:** Frequency of performance score instruments used in clinical trials

Instrument	Frequency
Karnofsky performance status	33
ecog (World Health Organization) performance score	11
General Performance Status	5
Study-designed performance instrument 22,23,40,50	4
Barthel index of activity of daily living	2

ecog = Eastern Cooperative Oncology Group.

**TABLE XIII:** Frequency of neurologic function instruments used in clinical trials

Instrument	Frequency
Neurologic function classification	21
Order classification	1
Study-designed neurologic instrument 71	1

**TABLE XIV:** Frequency of neurocognitive function instruments used in clinical trials

Instrument	Frequency
Mini Mental Status Examination	5
Controlled Oral Word Association test	2
Hopkins Verbal Learning Test	2
Trailmaking A and B	2
Grooved Pegboard	1
Mini Mental State Inventory (modified abbreviated version)	1
Ruff 2 and 7	1
